# Infection Prevention and Control Initiatives to Prevent Healthcare-Associated Transmission of SARS-CoV-2, East Africa

**DOI:** 10.3201/eid2813.212352

**Published:** 2022-12

**Authors:** Danica J. Gomes, Carmen Hazim, Jacqueline Safstrom, Carolyn Herzig, Ulzii Luvsansharav, Cori Dennison, Yakob Ahmed, Evelyn Wesangula, Joseph Hokororo, Jackson Amone, Berhanu Tekle, George Owiso, Rita Mutayoba, Mohammed Lamorde, Evelyn Akello, Getachew Kassa, Beniam Feleke, Linus Ndegwa, Kokuhumbya Kazaura, Diriisa Musisi, Anand Date, Benjamin J. Park, Elizabeth Bancroft

**Affiliations:** US Centers for Disease Control and Prevention, Atlanta, Georgia, USA (D.J. Gomes, C. Hazim, J. Safstrom, C. Herzig, U. Luvsansharav, C. Dennison, A. Date, B.J. Park, E. Bancroft);; Ministry of Health of Ethiopia, Addis Ababa, Ethiopia (Y. Ahmed);; Ministry of Health of Kenya, Nairobi, Kenya (E. Wesangula);; Ministry of Health, Dar es Salaam, Tanzania (J. Hokororo);; Ministry of Health of Uganda, Kampala, Uganda (J. Amone);; ICAP at Columbia University, New York, NY, USA (B. Tekle);; ICAP at Columbia University, Addis Ababa (G. Kassa);; I-Tech Kenya, Nairobi (G. Owiso);; Amref Health Africa, Dar es Salaam (R. Mutayoba);; Infectious Diseases Institute, Kampala (M. Lamorde);; Makerere University School of Public Health, Kampala (E. Akello);; US Centers for Disease Control and Prevention, Addis Ababa (B. Feleke);; US Centers for Disease Control and Prevention, Nairobi (L. Ndegwa);; US Centers for Disease Control and Prevention, Dar es Salaam (K. Kazaura);; US Centers for Disease Control and Prevention, Kampala (D. Musisi)

**Keywords:** COVID-19, infection prevention and control initiatives, coronavirus disease, severe acute respiratory syndrome coronavirus 2, SARS-CoV-2, coronaviruses, viruses, respiratory infections, low-income countries, middle-income countries, healthcare facilities, healthcare workers, essential healthcare services, capacity building, healthcare-associated transmission, zoonoses, Tanzania, Ethiopia, Kenya, Uganda, East Africa

## Abstract

The coronavirus disease pandemic has highlighted the need to establish and maintain strong infection prevention and control (IPC) practices, not only to prevent healthcare-associated transmission of SARS-CoV-2 to healthcare workers and patients but also to prevent disruptions of essential healthcare services. In East Africa, where basic IPC capacity in healthcare facilities is limited, the US Centers for Disease Control and Prevention (CDC) supported rapid IPC capacity building in healthcare facilities in 4 target countries: Tanzania, Ethiopia, Kenya, and Uganda. CDC supported IPC capacity-building initiatives at the healthcare facility and national levels according to each country’s specific needs, priorities, available resources, and existing IPC capacity and systems. In addition, CDC established a multicountry learning network to strengthen hospital level IPC, with an emphasis on peer-to-peer learning. We present an overview of the key strategies used to strengthen IPC in these countries and lessons learned from implementation.

Good infection prevention and control (IPC) practices are critical for preventing of healthcare-associated infections, maintaining essential healthcare services, and protecting patients and healthcare workers (HCWs) (*1**–**3*). Healthcare-associated infections can lead to poor clinical outcomes, more illnesses and deaths, longer hospital stays, and increased healthcare expenditures (*4**,**5*).

In addition to these negative effects of poor IPC practices on routine healthcare delivery, devastating consequences have also been highlighted during infectious disease outbreaks, in which healthcare facilities can serve as amplification points (*6**,**7*). As demonstrated by the COVID-19 pandemic and outbreaks of Ebola virus disease in West Africa and the Democratic Republic of the Congo, healthcare-associated transmission of infectious pathogens can lead to reductions in the healthcare workforce and a decrease in healthcare use because of safety concerns (*8**–**10*).

The COVID-19 pandemic showed gaps in IPC capacity globally and highlighted the need to build and reinforce national-, subnational-, and facility-level IPC programs aimed at protecting HCWs, patients, and visitors and preventing disruptions to essential healthcare services (*11*). Rapid capacity building becomes imperative during outbreaks such as COVID-19. However, building sustainable IPC systems and establishing good IPC practices at the healthcare facility level is a stepwise process that requires time and effort through multimodal approaches (*12*). Through platforms such as the United States President’s Emergency Plan for AIDS Relief (PEPFAR) and Global Health Security Agenda (GHSA), past US Centers for Disease Control and Prevention (CDC) investments and relationships have supported many countries and healthcare facilities in navigating this process. These efforts aimed to ensure a trained and dedicated IPC workforce that had adequate resources and guidelines to successfully implement IPC programs (*13*).

In East Africa, where basic IPC capacity in healthcare facilities is limited, CDC leveraged existing platforms and built upon ongoing IPC efforts to provide technical assistance and funding support to 4 countries (Kenya, Uganda, Ethiopia, and Tanzania) to strengthen IPC and reduce healthcare-associated transmission of SARS-CoV-2. We present an overview of the strategies CDC used to support IPC capacity building in these 4 countries during the pandemic, as well as lessons learned from implementation.

## CDC Contributions

In each country, CDC collaborated with the Ministry of Health and implementing partners to identify IPC gaps and priorities, and to develop tailored work plans to rapidly build capacity in priority areas, expand existing IPC initiatives, and strategically plan and implement COVID-19 prevention activities (Table). Different approaches and interventions for IPC strengthening were used across the 4 countries according to their specific needs, priorities, available resources, and existing IPC capacity and systems.

**Table:** Key infection prevention and control initiatives supported by CDC in response to the COVID-19 pandemic by country, 2020–2021*

Country	National level				Healthcare facility level			
	Develop national IPC unit	Develop guideline, policy, strategic plan	Develop monitoring and evaluation framework		Establish and develop IPC focal persons and committees	Develop COVID-19‒specific IPC guidance and interventions	HCW training and mentorships	Assess and monitor IPC practices
Kenya	Supported existing IPC unit	X	‒		X	X	X	X
Uganda	X	X	X		X	X	X	X
Ethiopia	X	X	‒		X	X	X	X
Tanzania	Supported existing IPC unit	X	X		X	X	X	X

*Information indicates activities supported by CDC during COVID-19 responses. This list is not comprehensive of IPC activities and resources available in each respective country. CDC, Centers for Disease Control and Prevention; IPC, infection prevention and control; X, CDC supported the respective activity in response to the COVID-19 pandemic; ‒, CDC did not support the respective activity as part of the response to the COVID-19 pandemic.

Commonly identified gaps across the 4 countries included limited IPC programs at the national, subnational, and healthcare facility levels; limited IPC knowledge and practices among HCWs; shortages of personal protective equipment (PPE); and inadequate infrastructure (e.g., sanitation and hygiene facilities, ventilation). Planned and implemented activities aligned with CDC’s operational considerations to prevent COVID-19 transmission in non-US healthcare settings and World Health Organization core components. The core components provide evidence-based recommendations on strengthening IPC programs and practices at the national, subnational, and facility levels (*12**–**14*). To further enhance IPC capacity building across the region, CDC supported establishing a multicountry learning network to cultivate hospital-level IPC capacity building, with an emphasis on peer-to-peer learning.

## Supporting National IPC Programs

To properly strengthen and sustain IPC across a healthcare system, national IPC governance structures are needed with the authority, expertise, and resources to oversee IPC programs, strategic plans, policies, and reporting mechanisms (*15*). On the basis of the strengths and gaps of each country, CDC supported capacity building at the national level by helping to establish and strengthen the national IPC unit, developing national IPC strategic plans, policies, and COVID-19 specific IPC guidelines, and creating an IPC monitoring and evaluation framework.

### Supporting National IPC Units

Personnel with dedicated IPC training, time, and resources are key to ensuring prioritization of IPC and sustained improvements. Before the COVID-19 pandemic, all 4 countries in East Africa had a designated IPC focal person at their respective Ministries of Health. Only Kenya and Tanzania had formal, well-established national IPC units with dedicated staff, budgets, and strategic action plans. To rapidly strengthen national capacity to address the COVID-19 pandemic and implement national IPC priorities, CDC provided resources for the Ministries of Health in Uganda and Ethiopia to hire staff for national IPC units.

### Development of Guidelines, Policies, and Strategic Plans

IPC guidelines establish standards for local adherence and guide healthcare facility leadership and HCWs in proper implementation of routine activities and strategic initiatives. National IPC policies and strategic plans are essential because they ensure alignment of national priorities and implementation efforts (*12*). National IPC guidelines were in place in each of the 4 countries before the pandemic. CDC investments and initiatives in Kenya before the pandemic were instrumental in the development of guidelines, national IPC strategic plans, and policies.

As part of IPC capacity building efforts in response to the COVID-19 pandemic, CDC supported development of the first national IPC strategic plan and policy documents for Ethiopia, which was linked to establishment of the national IPC unit. Similarly, technical assistance was provided to Tanzania in developing a 5-year plan for strengthening IPC. In Uganda, CDC is supporting the development of a national IPC strategic plan.

Regarding IPC guidelines, CDC supported development of national standard operating procedures for COVID-19 specific case management and IPC in Tanzania and is assisting with upcoming revisions of national guidelines for Uganda. Moreover, CDC supported Uganda in drafting national guidelines for managing COVID-19, which included content on HCW monitoring and management, screening and triage, rational use of PPE, waste management, and environmental cleaning and disinfection.

### Monitoring and Evaluation

Monitoring IPC indicators at the national and healthcare facility level is essential to understanding whether IPC standards and priorities are being met, identifying gaps, and informing necessary improvements and interventions (*16**,**17*). CDC supported the national IPC unit in Tanzania in development of a national IPC monitoring and evaluation framework and protocol, including key IPC performance indicators for central reporting, which involved collaborating with national and international partners. Follow-up activities will include disseminating the monitoring and evaluation framework to healthcare facilities across Tanzania and orienting key HCWs to its contents and reporting methods. Likewise, in Uganda, CDC is supporting the development of an IPC monitoring and evaluation framework with key performance indicators to strengthen IPC program implementation, monitoring, and evaluation in the country.

## Facility-Based IPC Initiatives

CDC collaborated with Ministries of Health and implementing partners and used the PEPFAR platform to rapidly build capacity in healthcare facilities across the 4 supported countries. Although initiatives aimed to construct and strengthen facility-level IPC programs, key areas of focus to prevent healthcare-associated SARS-CoV-2 transmission included establishing appropriate screening and triage of patients upon initial encounter at a healthcare facility to ensure early identification and isolation of patients with suspected COVID-19, limit ingthe entry of HCWs and visitors with suspected COVID-19, and identifing and isolating inpatients with suspected COVID-19. However, in Tanzania, in alignment with Ministry of Health priorities, efforts were focused on strengthening basic IPC and preventing all healthcare-associated infections. Efforts also included environmental controls to minimize crowding and ensure adequate ventilation in patient care and waiting areas.

Overall, Ethiopia has ≈4,500 public hospitals and health centers and 16,000 health posts; Tanzania has ≈9,800 healthcare facilities, including dispensaries (*18*); Kenya has ≈14,000 facilities (*19*); and Uganda has ≈7,900 facilities. In each country, healthcare facilities were targeted to receive technical assistance and implementation support: 16 in Ethiopia, 73 in Tanzania, 238 in Kenya, and >2,000 in Uganda received IPC mentorship. The rationale and methods for selecting facilities to support varied across countries. Facilities were prioritized on the basis of factors such as geographic location, patient volume, baseline capacity as determined by readiness assessments, existing relationships with implementing partners, or whether facilities were receiving ongoing CDC investments through existing platforms (e.g., PEPFAR). All countries ensured facilities targeted for support from CDC did not receive duplicative support from other nongovernmental organizations.

### IPC Focal Persons and Committees

CDC efforts aimed to ensure that healthcare facilities had functional IPC focal persons and committees by building the capacity of each individual to lead and oversee IPC activities in their respective facilities. In Uganda, implementing partners supported capacity building for existing IPC focal persons and establishment of new IPC focal persons and committees at facilities across 59 of 145 districts in Uganda. The Ministry of Health in Tanzania, through collaborations between CDC and implementing partners, improved existing IPC committees and focal persons in 73 healthcare facilities across 7 of the 31 regions in Tanzania by cascading a train-the-trainer strategy extending from national level IPC experts to regional and district teams to facility-based IPC focal persons and committees. In Ethiopia, where CDC and implementing partners focused heavily on COVID-19‒specific interventions, baseline assessments found that, in the 16 supported healthcare facilities, IPC focal persons were in place but IPC committees, if they existed, were often inoperative. Likewise, in Kenya, IPC facility readiness assessments revealed that many county-level IPC focal persons were not functional. To address these gaps, training and mentorship for IPC focal persons and committees was conducted. In Ethiopia, checklist tools were also created for focal persons and committee members to support proper performance of routine tasks.

### Development and Implementation of COVID-19‒Specific IPC Guidance and Processes

In the context of novel pathogens, developing targeted IPC guidance and protocols for implementation is crucial to communicating recommended IPC standards to frontline HCWs, guiding IPC practices, and ensuring safe delivery of essential healthcare services. Although guidance on COVID-19 prevention was made available in these countries, many healthcare facilities lacked standard operating procedures for implementation of COVID-19‒specific IPC activities. In Ethiopia, CDC and implementing partners developed standard operating procedures and tools to guide facilities in conducting screening and triage for patients and visitors, HCW screening and monitoring, and identifying and cohorting inpatients. As of July 2021, all 16 supported healthcare facilities were conducting COVID-19 screenings for arriving patients and visitors. In addition, in the 9 facilities with inpatient services, patients with suspected COVID-19 were isolated. In Kenya, systems for screening, triage, and patient isolation were implemented in 238 healthcare facilities across 13 priority regions through quality improvement processes. To improve early recognition and isolation of inpatients and HCWs who had suspected COVID-19, Kenya prioritized establishing inpatient surveillance and HCW monitoring in a smaller cohort of facilities. As a result, a system for inpatient surveillance was started at 10 hospitals.

Implementing partners and CDC staff in Ethiopia spearheaded facility mapping to develop schematics of existing patient and visitor flow. Information gathered was used to reengineer the internal patient and visitor flow, incorporate screening stations for patients and visitors, separate screening stations for HCWs, and establish waiting and testing stations for persons with respiratory symptoms or infections. Similar activities were conducted in Kenya and Uganda.

Last, in Ethiopia, efforts also focused on reinforcing appropriate hand hygiene practices. These practices were addressed by increasing hand hygiene stations throughout facilities and using quality improvement measures to bolster compliance.

### HCW Training and Mentorship

Training and mentorship were core methods used in educating HCWs and ensuring proper adherence to IPC guidance and recommended practices. In Kenya, implementing partners provided technical assistance to frontline HCWs and focused on effectively establishing standard, droplet, and airborne precautions. In addition to these COVID-19 prevention activities, training addressed rational use of PPE by using videos and demonstrations on donning and doffing. Furthermore, facility-level mentorship focused on establishing an appropriate triage process, ensuring adherence to recommended isolation practices, and instituting HCW and inpatient monitoring for COVID-19. Finally, biweekly IPC webinars were established to build IPC capacity at the facility level.

In Ethiopia, implementing partners trained HCWs on standard and transmission-based precautions, as well as COVID-19‒specific IPC interventions. More than 200 HCWs received comprehensive IPC training for COVID-19 and >3,200 ancillary staff received job-specific IPC training for COVID-19. To promote good mentorship and supportive supervision practices, the regional health bureaus developed tools and checklists for mentors. Emphasis was placed on the need for performance indicators to guide improvement plans.

In Uganda, leveraging a mentorship approach developed to build IPC capacity during the 2018–2020 Ebola virus disease outbreak in the Democratic Republic of the Congo, CDC supported implementing partners in providing mentorship to >2,000 healthcare facilities across 59 districts. Mentorship focused on addressing COVID-19‒specific IPC, including screening, triage, and isolation; standard and transmission-based precautions; risk assessment; and work plan development. CDC provided technical support for evaluating the IPC mentorship program; the evaluation results will strengthen the approach and inform next steps of the IPC mentorship program to sustain improvements and address lingering gaps.

In Tanzania, IPC focal persons trained by regional and district teams mentored and cascaded general IPC training to >700 frontline HCWs. Trainings were focused on screening and triage for all infectious diseases and general IPC topics, such as standard-based and transmission-based precautions, medical device disinfection and sterilization, waste management, prevention and surveillance of key healthcare-associated infections, and antimicrobial drug resistance. After those comprehensive trainings, facility-based IPC focal persons provided daily IPC mentorship to frontline HCWs during routine job-related activities.

### Assessment and Monitoring of IPC Practices and Activities

IPC assessments to identify gaps and determine priorities are essential to guide IPC interventions and inform the development of tailored workplans. Equally necessary is the routine monitoring of IPC practices to ensure effectiveness and guide needed adjustments to IPC improvement strategies.

In Ethiopia, baseline and monthly IPC assessments determined the level of IPC readiness among CDC-supported healthcare facilities. The assessment tool was used to collect data on core elements aimed at preventing healthcare-associated SARS-CoV-2 transmission. Initial results, in December 2020, showed that most facilities lacked procedures, training, designated spaces, supplies, and equipment for patient screening and triage, HCW screening, and inpatient isolation and cohorting. Among 11 facilities, only 3 facilities had initiated patient screening and triage, and none had started screening HCWs for COVID-19. These findings informed the development of site-specific workplans, which were put in place by facility-based IPC focal persons in collaboration with implementing partners. HCWs received targeted COVID-19 training; close mentorship and support for IPC implementation; and the necessary supplies, equipment, and space reorganization for compliance with interventions for preventing SARS-CoV-2 transmission in the healthcare facilities. After 1 year, all facilities had patient screening and triage systems in use and were actively conducting COVID-19 screening for HCWs. Likewise, facilities with inpatient services had started inpatient isolation and cohorting procedures.

CDC supported the completion of comprehensive IPC assessments in targeted healthcare facilities in Tanzania by using the nationally approved Standards-Based Management and Recognition IPC assessment tool. IPC assessment results are fed back to the national level for review to guide IPC capacity building decisions and efforts at the national level.

In Uganda, CDC supported development of a facility-level IPC assessment tool that was used to monitor IPC improvements across facilities in which IPC mentorships were conducted. Data collected were analyzed to inform ongoing IPC programming.

A national healthcare facility assessment for COVID-19 IPC was conducted rapidly in all 47 counties in Kenya by using mobile applications, and results were used for rapid planning and resource mobilization. A triage monitoring checklist, developed by CDC, was used to audit and collect data on the screening and triage activities in the 238 supported healthcare facilities. With CDC and implementing partner support, healthcare facilities targeted in Kenya also focused efforts on monitoring and ensuring the appropriate use of PPE, with specific attention to mask use during healthcare delivery.

## East Africa IPC Network

The East Africa Infection Prevention and Control Network establishes a regional IPC Community of Practice; supports training, capacity building, knowledge sharing and joint learning; and implements quality improvement projects. The main goal of this initiative is to reduce the incidence of COVID-19 and healthcare-associated infections by improving compliance with IPC standards at participating hospitals. The network comprises 22 hospitals across the 4 countries: Ethiopia (5 hospitals), Kenya (6 hospitals), Tanzania (5 hospitals), and Uganda (6 hospitals). The Ministry of Health of each country, the International Center for AIDS Care and Treatment Programs at Columbia University, and CDC worked together to select participating hospitals.

The East Africa Infection Prevention and Control Network learning activities include weekly case-based learning sessions, monthly webinars, and trainings on quality improvement methods and science. Activities focus on professional development for facility IPC focal persons who receive direct, in-person supportive supervision from local IPC mentors. This hands-on support enables an exchange of best practices; skills building; innovation; and rapid dissemination of tools, case studies, and implementation strategies. Moreover, a regional IPC advisor provides oversight and support to all 4 countries. Topics covered in learning sessions, webinars, one-on-one mentorship, and supplemental trainings are prioritized on the basis of results from IPC focal person self-assessments and facility IPC assessments. The network also supports a moderated Telegram group (instant messaging communication platform) to share documents and resources, conduct polling, and connect IPC focal persons across the region with one another to improve communication.

## Challenges to Improving IPC and Lessons Learned

Many challenges were encountered in working to improve IPC in these complex settings during the COVID-19 pandemic. First, countries had varying degrees of established national-, subnational-, and facility-level IPC programs, and existing surveillance and prevention activities needed strengthening. However, having an infrastructure of dedicated staff was still critical to quickly ramping up in the face of a large pandemic. Ensuring good coordination and communication between Ministries of Health, implementing partners, and frontline HCWs and facilities was critical. Continued support of these structures will be essential as the pandemic evolves and for emerging threats to healthcare delivery. Moreover, prioritizing IPC at the national level requires commitment from leadership and resource allocation to ensure sustainable capacity building over time.

The global shortage of PPE presented an additional challenge to healthcare facilities because most facilities did not have a reliable PPE supply chain or system to monitor PPE use and stock. There is a need to improve the international and national supply chains for PPE, implement systems for monitoring PPE use and stock, and ensure correct and rational use of PPE by HCWs.

Although lack of PPE was a major IPC gap, provision of PPE alone was not enough to improve IPC practices. Standardized IPC protocols were often lacking and, even when in place, adherence was limited. Frontline HCWs had limited IPC knowledge, which necessitated a heavy emphasis on training and mentorship. However, onsite support to healthcare facilities and HCWs was challenged by COVID-19 restrictions. Much of the communication took place through virtual platforms, but internet connections were not always reliable and interfered with the ability to provide remote technical assistance. As access to technology improves, so will opportunities for online education, virtual technical assistance, and even remote evaluations of IPC at healthcare facilities.

Surveillance for COVID-19 among HCWs and patients was challenging. Early in the pandemic, HCW monitoring for COVID-19 was not prioritized. Surveillance for new symptoms of COVID-19 among inpatients also proved difficult because few facilities had a system for routine identification of healthcare-associated infections. Therefore, new surveillance paradigms had to be created and implemented. For example, CDC, in collaboration with in-country partners and Ministries of Health, was able to quickly launch facility-based monitoring tools that are being used to track effect in facilities. Data to ascertain the relevance of these indicators are needed to inform future IPC capacity building strategies and implementation efforts.

## Conclusions

In East Africa, the COVID-19 pandemic showed major gaps in healthcare facility IPC that needed to be addressed to preserve the safe delivery of essential healthcare services. Rapidly building IPC capacity emerged as a key priority to stemming the spread of COVID-19, and leveraging existing platforms (e.g., PEPFAR, GHSA) contributed to rapid implementation of IPC interventions. As a result of IPC improvement initiatives, IPC programs were established or strengthened at national and healthcare facility levels; IPC focal persons and committees were put in place and capacitated; IPC guidance, national strategic plans and policies, and monitoring and evaluation frameworks were developed; HCWs received IPC training and mentorship; IPC assessments of healthcare facilities were conducted; and, informed by results of IPC assessments, quality improvement interventions were put into place.

Although these interventions materialized in response to COVID-19, many were based on work started before the pandemic to support long-term, sustainable IPC improvement efforts at the healthcare facility and national level. To reduce routine healthcare-associated infections and avert future outbreaks, interventions implemented to achieve a rapid expansion of IPC and reduce the spread of SARS-CoV-2 in healthcare facilities should be sustained and expanded to reduce transmission of other endemic infectious diseases, including tuberculosis and influenza, and other respiratory and nonrespiratory pathogens. Countries, donors, and implementing partners should build upon programs developed for COVID-19 to improve healthcare safety beyond the pandemic. Although IPC funding might decrease as the pandemic subsides, continued prioritization of IPC by Ministries of Health and national IPC leaders within each country can result in continued progress and momentum with regard to IPC strengthening.
